# The State-of-the Art of Personalized Vaccines for Non-Communicable Diseases: A Narrative Review

**DOI:** 10.3390/vaccines14080693

**Published:** 2026-08-12

**Authors:** Mario Caldarelli, Pierluigi Rio, Carlotta Renna, Andrea Marrone, Giulia Guazzarotti, Antonio Gasbarrini, Giovanni Gambassi, Rossella Cianci

**Affiliations:** 1 Department of Translational Medicine and Surgery, Catholic University of Sacred Heart, 00168 Rome, Italy; mario.caldarelli01@icatt.it (M.C.); pierluigi.rio01@icatt.it (P.R.); carlotta.renna@guest.policlinicogemelli.it (C.R.); andrea.marrone01@icatt.it (A.M.); giulia.guazzarotti01@icatt.it (G.G.); antonio.gasbarrini@unicatt.it (A.G.); giovanni.gambassi@unicatt.it (G.G.); 2 Fondazione Policlinico Universitario A. Gemelli, Istituto di Ricerca e Cura a Carattere Scientifico (IRCCS), 00168 Rome, Italy

**Keywords:** personalized vaccines, therapeutic vaccines, non-communicable diseases, cancer, neoantigens, immune tolerance, mRNA vaccines, immunotherapy, precision medicine

## Abstract

Non-communicable diseases (NCDs), including cancer, cardiovascular, neurodegenerative, autoimmune, and allergic diseases, account for a significant amount of global morbidity and mortality. Chronic viral infections have not been recognized as NCDs, despite the availability of several therapeutic vaccination strategies against oncogenic viruses, such as the hepatitis B virus (HBV) and the human papillomavirus (HPV). Indeed, chronic viral infection leads to the development and progression of malignancies directly linked to the viruses. These considerations support a connection between NCDs, infectious diseases, and therapeutic vaccination. Recent advances in technology have paved the way to the use of vaccines beyond the prevention of infectious diseases, heralding innovative therapeutic and preventative strategies for a variety of chronic NCDs. Here we will review the state of the art of personalized vaccine strategies for NCDs, with an emphasis on the diverse technological platforms used to develop them, including mRNA and DNA vaccines, viral vectors, dendritic cell-based vaccines, nanoparticle delivery systems, and next-generation adjuvants. The review intends to make the case for personalized and antigen-specific vaccination strategies as a compelling option for precision immunotherapy primarily in oncology, where neoantigen-based vaccines are being developed. In addition, we will also review tolerogenic vaccination strategies, vaccination strategies targeting pathological proteins and pathways in neurodegenerative and cardiovascular diseases, and vaccine-based treatment of chronic viral infections. Current evidence about vaccines suggests that several ways are available to induce an immune response or create tolerance to a disease, and possibly altering its course instead of simply controlling the symptoms. However, many challenges are still to be overcome, including disease variability, how to identify appropriate target antigens, the complexity of manufacturing process, long-term safety, and integration with already established treatments. By virtue of the convergence of multiple fields—immunology, genomics, bioinformatics, and new delivery systems—precision vaccinology is gaining momentum. Thus, it is envisaged that personalized vaccines will be an increasingly essential component of future preventive and therapeutic approaches for non-communicable diseases.

## 1. Introduction

A non-communicable disease (NCDs) has a chronic character, and no link to acute infectious processes can be evoked. Cancer, cardiovascular diseases, neurodegenerative and autoimmune diseases, and allergies are among them. In contrast, chronic viral infections are considered separately from NCDs.

Despite significant progress in the development of effective pharmacologic and biologic therapeutics to treat cancer, autoimmune, cardiovascular, neurodegenerative diseases, and chronic viral infections, NCDs continue to account for most of the global morbidity and mortality. Historically, treatment of NCDs has been aimed primarily at controlling symptoms, delaying disease progression, and targeted molecular therapies. However, these traditional approaches are often of limited effectiveness, resistance to treatment and the emergence of side effects are common and, after all, these approaches cannot restore a physiological homeostatic state. For such reason, new therapeutic paradigms are emerging which aim at achieving a durable disease modification by stimulating the immune system. Personalized vaccination strategies are necessary given the biological variability of NCDs. Individuals who share the same clinical diagnosis can have fundamentally different molecular mechanisms for their disease, as well as diverse antigenic landscape, immune cell types, and in the end a unique treatment response. Vaccines have been primarily adopted for the prevention of infectious diseases, and they have marked historic public health milestones. Nowadays, scientific advances have expanded the potential of vaccine technology beyond infectious pathogens and offer new and exciting possibilities for both prevention and treatment of NCDs [1].

Therapeutic and personalized vaccines are fundamentally different from preventive vaccines as they are created to activate the immune system depending on the specific cause of the disease. In this respect, a vaccine is intended to elicit immune reactions against pathogens or to induce tolerance to antigens in patients with autoimmune or other chronic illnesses. In the case of other NCDs, vaccination can activate protective mechanisms or neutralize harmful elements in the body. Therefore, vaccines can have either immunostimulatory, tolerogenic, or immunomodulatory properties depending on the case [2,3].

The introduction of novel vaccine technologies—including mRNA, DNA, self-amplifying RNA, viral vector, dendritic cell-based vaccines, nanoparticles, virus-like particles, and exosome-based systems—now allows more precise control of antigen delivery, of immune response activation, and of tissues targeting [4]. The accelerated use of mRNA vaccines in clinical practice during the COVID-19 (COronaVIrus Disease 2019) pandemic has also fueled the application of the same technologies to patients with complex chronic diseases [5].

Yet, a more widespread use of personalized vaccination is still facing difficulties. Important remaining challenges are the identification and prioritization of specific antigens to employ; the constraints of the manufacturing process; various quality control processes; the necessity of estimate potential risks related to vaccination; the different immune responses evoked in each person; and the best timing for immunization.

Among all NCDs, cancer is the most promising candidate in the field of personalized vaccine development, primarily because of the availability of tumor-specific neoantigens, tumor tissue sequencing, and established strategies to stimulate cytotoxic T-cell responses. In contrast, vaccine development for neurodegenerative and cardiovascular diseases is only at an early stage and faces additional challenges related to endogenous antigen targeting, chronic inflammation, tissue accessibility, and the need to avoid autoimmune reactions. Beyond the more nuanced disease-specific differences, personalization refers to the integration of next-generation sequencing, transcriptomics, proteomics, immunopeptidomics, advanced immune profiling, and bioinformatics tools, including artificial intelligence-based algorithms, to identify patient-specific antigens to guide vaccine design. Consequently, personalized vaccination entails not only the selection of an appropriate technological platform, but requires the identification of individual antigenic targets and longitudinal monitoring of immune responses, configuring the development of precision vaccines as very different from conventional ones [2,6].

Beyond oncology, new reverse-vaccination strategies are being explored for patients with autoimmune diseases, and new vaccines against endogenous antigens have demonstrated promising results in clinical trials in patients with neurodegenerative and cardiovascular diseases [7,8]. In this narrative review, we aim to summarize the rapidly evolving field of personalized vaccines for the treatment of NCDs. To this end, modern technology for the development of new vaccines will be summarized, and we will review data about the use of existing vaccines for some types of cancer, in autoimmune diseases, neurologic, heart and circulatory system disorders, and in chronic viral related diseases. We searched PubMed/MEDLINE and Google Scholar using various combinations of ‘personalized vaccines’, ‘therapeutic vaccines’, ‘tolerogenic vaccines’, ‘antigen-specific immunotherapy’, ‘neoantigens’, ‘mRNA vaccines’, ‘DNA vaccines’, ‘viral vectors’, ‘dendritic cell vaccines’, ‘nanoparticles’, and ‘cancer’, ‘autoimmune diseases’, ‘neurodegenerative diseases’, ‘cardio-vascular diseases’, ‘allergic diseases’, ‘hepatitis B virus’, and ‘human papillomavirus’. We focused on peer-reviewed articles published after 2015 and included only some ground-breaking articles prior to that date if they were deemed essential for understanding historical evolution. Priority was given to original studies, at both preclinical or clinical stages, but some reviews were also used to capture other relevant studies. Special emphasis was placed on studies such as randomized clinical trials and late-phase studies. A formal systematic review protocol, pre-defined eligibility requirements, or a quantitative meta-analysis were not utilized in this review. Evidence was synthesized qualitatively, distinguishing three types of study: preclinical proof-of-concept studies, early-phase clinical studies focused on safety and immunogenicity, and advanced clinical trials evaluating clinical efficacy.

## 2. Technological Platforms and Design Strategies for Vaccines in NCDs

Developing a personalized vaccine involves a combination of antigen discovery, vaccine platform choice, patient stratification, and individualized immune monitoring. Unlike conventional vaccines that deliver a single antigen formulation to an entire population, personalized vaccines are tailored to the specific molecular, genetic, and immunological profile of a single patient or of a relevant clinical subgroup. The development process includes genomic and transcriptomic analysis, immunopeptidomic analysis, computation-based antigen predictions, and artificial intelligence-based prioritization of candidate targets, as well as the selection of the most appropriate delivery platform. Thus, personalization can occur at many levels: from the target antigen to the antigen sequence, vaccine formulation, and delivery system.

To approach the development of personalized vaccines for NCDs is of paramount importance the definition of the biological aim. For example, in the case of cancer it is possible to customize the vaccine based on the tumor or individual antigens—including neoantigens—due to somatic mutations. Differently, autoimmune diseases need vaccines that are designed to restore immune tolerance, while in the case of cardiovascular and neurodegenerative diseases, vaccines are aimed at achieving immunoprotective antibody responses, at neutralizing harmful proteins, or at controlling chronic inflammation. Therefore, a specific type of technology can be utilized for different aims depending on the type of disease [9].

Antigen-based vaccines remain widely used to stimulate the immune system to initiate a targeted response against defined antigens associated with disease. However, the clinical effectiveness of these antigen-based vaccines is often limited by low intrinsic immunogenicity and by the need for highly effective adjuvants to enhance responsiveness. On the other hand, nucleic acid-based vaccine platforms (DNA or mRNA) are highly versatile systems capable of eliciting robust humoral and cellular immune responses. Depending on the targeted antigen, formulation, and route of administration of mRNA vaccines, it is possible to elicit both cellular and humoral immune response, although the relative contribution of CD8+ T cells, CD4+ T cell subsets, and the antibody response varies with the vaccine design and with the specific disease.

The development of mRNA vaccines in oncology is especially noteworthy due to their rapid large-scale manufacturing combined with their strong immunogenicity [1].

One form of vaccine uses a viral vector, either adeno- and/or poxvirus, to enhance efficiency of antigen presentation, as well as to improve priming of T-cells. Another type of vaccine is cellular and utilizes dendritic cells (DCs) loaded with autologous antigens to create strong T-cell responses against specific antigens [10].

Novel modalities of vaccine delivery now include lipid nanoparticles and next-generation mRNA vaccine constructs containing an adjuvant are improving antigen stability, delivery to the immune system, as well as T-cell responses and have demonstrated antitumor efficacy in preclinical studies. Altogether, the initial studies seem to indicate that a precision approach to immunization is feasible and that is of potential benefit in both prevention and treatment of NCDs [6]. The main vaccine platforms, along with their underlying mechanisms, advantages, and current limitations, are summarized in Table 1.

In oncology, tumor-associated antigens (TAAs) and, more importantly, tumor-specific neoantigens generated by somatic mutations enable the design of highly individualized vaccines tailored to each patient’s mutational profile. Recent studies have shown that neoantigen-based mRNA vaccines can induce durable CD4+ and CD8+ T-cell responses and long-term immunological memory, particularly when combined with immune checkpoint inhibitors such as anti-PD (Programmed cell death protein)-1/PD-L1 antibodies, while next-generation sequencing and bioinformatic pipelines have markedly improved the speed and accuracy of neoantigen identification, despite remaining important clinical bottlenecks [2]. Personalization in oncology is therefore supported by whole-exome and whole-genome sequencing, transcriptomic profiling, immunopeptidomics, and computational prioritization of candidate epitopes according to mutation expression, HLA (human leukocyte antigen) binding, antigen processing, and predicted immunogenicity, allowing iterative refinement of vaccine targets as tumors evolve under immune pressure. In contrast, vaccine development for non-oncological NCDs has largely focused on targeting self-antigens involved in disease pathogenesis, such as ApoB in atherosclerosis or misfolded proteins including amyloid-β and tau in neurodegenerative disorders, with the objective of generating protective or regulatory immune responses while preserving immune tolerance and minimizing the risk of autoimmune complications [3,7,11].

From a technological perspective, advancements need to be made to improve the mechanisms of antigen presentation and the relative immune activation. An example of a potential strategy is the use of self-amplifying RNA (saRNA) or circular RNA (circRNA) platforms to enhance antigen expression time and increase translational efficiency compared to traditional mRNA. These platforms enable dose reduction and have the potential to enhance the magnitude and duration of immune responses, particularly when prolonged exposure to an antigen is necessary. Furthermore, advances in codon optimization, untranslated region engineering, and nucleoside modification are also improving RNA stability and decreasing innate immune detection, which could otherwise decrease translational output [5]. However, there are also numerous emerging vaccines that utilize newer technology in the form of synthetic or semi-synthetic lipid nanoparticles with added features like ionizable lipids (although still in pilot trials), targeting ligands, and stimuli-responsive elements to enhance the amount of accurately represented antigen taken up by cells and escaped from endosomes [12]. Likewise, innovative methods of vaccine delivery are also being developed. Examples include polymeric nanoparticles, virus-like particles (VLPs), and exosome-like materials that allow antigen packaging and presentation while limiting toxicity. VLPs, for example, are capable of presenting repetitive types of antigens in a way that is highly immunogenic and facilitate cross-linking of B-cell receptors, while exosome-like vaccines use the ability of cells to communicate with each other to deliver antigens in a physiological manner [13].

Adjuvants play the role of modulating innate immune sensing and stimulating the adaptive response to activate immunity response induced by vaccines. There are several types of adjuvants that act on various pattern-recognition receptor (PRR) pathways to contribute to the process of cytokine synthesis, maturation of antigen-presenting cells, and T-cell and B-cell responses. However, the immune-related effects attributed to an adjuvant depend upon a combination of activated receptor pathways, cellular state, method of infusion, and strength and length of stimulation of innate immunity. Hence, the choice of an adjuvant deserves critical attention while developing personalized vaccines since excessive activation of innate immunity may be harmful in tolerance therapy, while low activation can lead to lower efficiency of antitumoral killing [14]. It is noteworthy that there is an increasing number of cell-based, and ex vivo engineered therapeutic approaches available. In addition to vaccination with dendritic cells, novel therapeutic strategies include the use of engineered antigen presenting cells, artificial antigen presenting cell (APC) systems, and T cell targeted vaccinations aimed at directly activating cytotoxic immune response. Most of the platforms used also incorporate genetic modification techniques that support the optimization of antigen processing and presentation pathways, or alternatively change inhibitory signaling mechanisms of the immune response [15]. Finally, the route of administration of a vaccine, as well as the ability to target tissue specifically, are being increasingly recognized of critical importance. Recently, both intradermal and mucosal routes of administration have received attention because of their ability to access the immune cells in those tissues. Furthermore, advances in delivery systems, such as microneedle patches and implantable systems, have made it possible for vaccines to provide antigen controlled release and to improve patient compliance. All these technological advancements mark a shift from empirical vaccine designs to engineered immunotherapeutic platforms, where any aspect, antigen design, delivery, and immune modulation, is optimally selected [15].

Yet, there are still many obstacles to overcome before we can ensure every patient receives effective treatment. The main obstacle is the heterogeneity of NCDs which makes it difficult to identify common treatment targets for different patients. Immune senescence and other comorbidities influence the ability to respond to a vaccine. In addition, the need to consider combination strategies, where vaccines are to be used in conjunction with checkpoint inhibitors, cytokine therapy, and metabolic modulation therapies, has created more challenges than anticipated in the design and conduct of clinical trials. Nonetheless, the field of vaccines for NCDs has transitioned from a static ‘one-size-fits-all’ approach to a dynamic modular system wherein specific combinations of antigens, delivery systems, and immune modulation practices are purposefully utilized to develop preventive and therapeutic vaccination strategies targeting distinct disease-causing mechanisms. Despite these changes, the overall contribution and optimal use of these platforms vary across diverse disease settings due to differences in immunobiology, target accessibility, and intended therapeutic goal. In the following sections we will review how technological platforms may be differentially utilized and adapted across major NCD categories, highlighting similarities as well as challenges that are impacting how vaccine-based treatments are being studied and developed.

### Comparative Selection of Vaccine Platforms According to Therapeutic Objectives

When selecting the appropriate vaccine platform, it is important to base the decision on the required immunological outcome rather than solely on technological advancements. Platforms differ on antigen presentation, cross-presentation, stimulation of innate immunity, production of antibodies, administrability, and the possibility of inducing an immune response. Therefore, the best platform depends on the specific disease and the immunological outcome.

For inducing cytotoxic immunity against tumors, mRNA, DNA, viral vectors, dendritic cell vaccines, and a certain type of nanoparticles are most beneficial as they contribute to internal antigen presentation, effective MHC (Major histocompatibility complex) class I cross-presentation, and cytotoxic CD8+ T cell activation. mRNA platforms can provide additional advantage because of the faster design of antigens, which allows the development of individualized neoantigen vaccines, although their effectiveness depends on delivery efficiency and on the ability to overcome tumor microenvironment [16].

Protein and peptide vaccine types are preferred if the exact specifications of the antigen are of great importance, being essential for tolerogenic vaccination. Nevertheless, the success of such vaccines depends heavily on the choice of adjuvant, antigen processing pathway, and delivery system. Nanoparticle-based systems make antigen stability, lymphatic drainage, cellular uptake, and immunological signaling more efficient; however, these beneficial effects are affected by the composition and physicochemical properties of nanoparticles [17].

When it comes to tolerogenic use, the aim is to avoid too much innate immune activation. Thus, one needs to look for systems that provide controlled delivery of antigens without inducing strong co-stimulatory signal. These include nanoparticles, dendritic-cell tolerance-based systems, and targeted systems that utilize continuous delivery into the liver to stimulate anergy/deletion/regulation by T-cells and other mechanisms of antigen tolerance establishment.

The major personalized vaccination strategies across the principal non-communicable diseases, including their therapeutic targets, preferred vaccine platforms, immunological objectives, and current stage of clinical development, are summarized in Table 2. The following sections discuss these disease-specific applications in greater detail.

## 3. Tolerogenic Vaccination Strategies for Autoimmune Diseases

Tolerogenic vaccination strategies focus on self-antigens relevant to the disease that drive the body’s autoreactive T- and B-cell responses. Depending on the specific autoimmune disease, these antigens can include nucleic acids, associated antigens, and histones as in Systemic lupus erythematosus (SLE) or citrullinated proteins and antigens related to collagen in Rheumatoid Arthritis (RA), as well as myelin-related proteins in multiple sclerosis (MS). The goal is not to induce a total suppression of the immune system but to selectively restore tolerance to the antigen, hence eliminating the function of the pathogenic one while keeping the protective aspect of immunity intact. Autoimmune diseases represent a significant public health challenge, with 10% of the population in Europe suffering from at least one such condition. The incidence of autoimmune diseases is especially elevated in Northern Europe and demonstrates a growing trend across various age cohorts and geographic areas [20]. Autoimmune diseases result from the failure of immunological tolerance toward self-antigens, which triggers persistent immune responses and causes tissue-specific damage over time. Contemporary therapeutic approaches primarily focus on broad-spectrum immunosuppression as a means to mitigate inflammation and control disease activity [25]. Although these interventions are effective in alleviating symptoms and decelerating disease progression, they have substantial limitations. Broad-spectrum immunosuppressive therapies are associated with heightened vulnerability to severe infections and malignancies due to their non-specific targeting of the immune system. This lack of antigen specificity leads to unintended immune suppression beyond the intended targets. Notably, such treatments fail to address the fundamental breakdown in immune tolerance toward self-antigens, necessitating prolonged or lifelong usage to continue manage the disease [26]. Antigen-specific tolerogenic methods show great potential in inhibiting the body’s autoreactive lymphocytes while maintaining the immune response to other antigens. With the antigen being treated, such vaccine therapy can restore the immune tolerance instead of just eliminating inflammation. However, the main factor that defines the effectiveness of the treatment is the identification of the crucial autoantigen and the way of ensuring long-term tolerance without causing adverse autoimmunity [27]. Recent studies show that inverse vaccination strategies not only promote temporary immune suppression but also lead to lasting immunological changes through long-term reprogramming of autoreactive lymphocytes in a way that supports regulatory immune networks stability and function [28].

In autoimmunity, inverse vaccination promotes antigen-specific immune tolerance by inducing regulatory T and B cells and by favoring anti-inflammatory cytokine profiles in response to targeted autoantigen presentation [29]. There are several different mechanisms that can lead to antigen-specific tolerance. One of these mechanisms is T-cell anergy. T-cell anergy occurs when lymphocyte activation takes place without any co-stimulation signals being present. Another mechanism is clonal deletion. In this case, self-reactive T-lymphocytes are eliminated through apoptosis. Lastly, regulatory T cells may also be involved in the process of inducing antigen-specific tolerance. By doing so, they can influence the immune response using immunosuppressive cytokines and cell-to-cell interactions. Additionally, the action of tolerogenic antigen presenting cells triggers an immune non-inflammatory response to self-antigens. Finally, regulatory cells or molecules may induce an inhibitory immune response towards self-antigens [30]. A critical aspect of type II tolerance is the induction of regulatory T (Treg) cells through tolerogenic vaccination. Treg cells characterized by stable epigenetic signatures within the Forkhead box P3 (FOXP3) gene exhibit reduced phenotypic plasticity, limiting their ability to differentiate into pathogenic inflammatory effector T cells under inflammatory conditions [8]. This approach enables selective suppression of pathogenic immune responses while preserving systemic immune function.

The translation of these immunological principles into clinically applicable therapies has been facilitated by the development of advanced antigen delivery platforms. Induction of peripheral immune tolerance can occur through antigen presentation alone, without the activation of co-stimulatory signals, resulting in either T cell anergy or functional suppression [31]. Utilizing nanoparticle-based synthetic antigen-presenting cells allows for the presentation of antigenic epitopes without the presence of any co-stimulatory molecules that may generate the opposite effect (immunogenicity). This creates an environment conducive to tolerogenic immunology [32].

Beyond delivery systems that control antigen presentation at the cellular level, organ-specific targeting strategies have emerged to further exploit physiological mechanisms of immune tolerance. Recent studies are redefining our understanding of the induction of immune tolerance with the introduction of tolerogenic DCs and Tregs. These cells present antigen without first activating additional T cells through co-stimulation. During non-inflammatory processes, they rely on oxidative energy sources like fatty acid oxidation and electrons from the Krebs Cycle, instead of primarily using glycolysis [33].

The antigen-directed tolerogenic approach has proved effective in several animal models (including arthritis, Type 1 Diabetes (T1D) and MS). In preclinical type 1 diabetes models, regulatory T- and B-cell populations were induced using iron oxide nanoparticles carrying antigen-MHC class II complexes and disease onset was delayed or stopped [34]. The nanoparticles were also able to convert autoreactive T cells into regulatory-like T cells, which migrated to sites of inflammation, thereby inhibiting the subsequent activation of pathogenic T cells. The role played by the liver in the development and maintenance of adaptive immune tolerance by tolerogenic antigen presenting cells and through cytokine-mediated signaling is well established. Liver-directed antigen delivery promotes a type of trained tolerogenic immunity in innate immune cells, including hepatic macrophages and dendritic cells, by inducing stable epigenetic and metabolic reprogramming and also alters adaptive immune system [35]. On these bases, there has been an increased interest in the use of liver-targeted therapies, i.e., targeting autoantigens to the liver, to restore antigen-specific immune tolerance. In preclinical models, either hepatocyte-directed expression or delivery of relevant self-antigens for a particular autoimmune disease has been shown to generate antigen-specific FOXP3+ regulatory T cells, resulting in a decreased disease incidence and the reversal of pathological features [28,36]. Autoantigens conjugated to N-acetyl galactosamine (GalNAc) are preferentially taken up by the hepatic antigen-presenting cell via c-type lectin receptors and increase the effectiveness of tolerogenic antigen presentations. In mouse models of autoimmune diseases, GalNAc-conjugated autoantigens increase functional regulatory T cells, inhibit the proliferation of autoreactive T cells, and either prevent or reverse the development of autoimmune disease while exhibiting minimal systemic toxicity [37,38]. These antigen-specific tolerance–inducing strategies have been evaluated in multiple autoimmune diseases, providing disease-specific insights into their therapeutic potential. Interestingly, regulatory T cells developed in the liver have also the capacity to travel to other areas of the body (peripheral tissues) and suppress autoimmune responses not related to the original self-antigen (by using bystander suppression) [39].

SLE represents a prototypical model for evaluating antigen-specific immunotherapeutic approaches. SLE is a systemic autoimmune disease characterized by a lack of tolerance towards a wide range of nuclear and cytoplasmic autoantigens, including but not limited to double-stranded DNA, histones, and ribonucleoproteins. Autoreactive B and T cells play an important role in the formation of pathogenic autoantibodies and immune complexes, which in turn activate the complement system and innate immune response, leading to tissue inflammation. Immune complexes containing nucleic acids can activate endosomal Toll-like receptors and induce the production of type I interferons. Antigenic complexity and heterogeneity in SLE pose serious challenges to the development of antigen-specific tolerogenic vaccines [40,41]. Immunosuppressive medications and biologic treatments that inhibit B-cell survival pathways (such as by blocking B-lymphocyte stimulator) are the current therapeutic approaches used to treat SLE [42]. Despite a decrease in disease severity, these approaches typically result in chronic toxicity and do not promote immune reestablishment (restoration). In this respect, antigen-specific tolerogenic strategies are increasingly viewed as possible disease-modifying strategies capable of restoring immune homeostasis rather than merely suppressing disease activity [43]. Of all the antigen-specific methods studied for SLE, peptide-based methods have shown a great deal of promise in terms of inhibiting autoreactive immune responses without influencing the more general protective immunity. However, the mechanisms and targets of these methods are not the same [44]. One example of the immunomodulatory peptide approach is the peptide P140 from U1 small nuclear ribonucleoprotein 70 kDa protein (U1-70K) small nuclear ribonucleoprotein that aims to regulate T-cell responses and B-cell activity instead of just counteracting one pathogenic autoantibody. The use of P140 in different experimental models resulted in less damage to the organs affected by the disease, decreased levels of anti-dsDNA IgG, and better survival results [45]. The mechanism of action includes the regulation of MHC class II antigen presentation and autophagy-dependent antigen processing that influence CD4+ T-cell activation and consequent B-cell activity. Studies carried out on immune cells taken from SLE patients in the ex vivo setting indicated that this peptide might have an inhibitory effect on CD4+ proliferation and decrease production of IgG by B-cells [46,47]. Peptides that have been derived from histones represent a new and unique method of providing antigen-specific tolerance. In SLE, the presence of autoreactive T cells has allowed the identification of nucleosomal antigens that possess immunodominant epitopes within histones. This provides a basis for the use of epitopes in a tolerogenic immunotherapy system [48]. Among mice that demonstrate a tendency to develop lupus, the application of histone H471–94 delayed the onset of nephritis and improved survival versus the use of epitope peptides [49]. The properties associated with H471–94 are related to the immunomodulatory effect caused by the reduced production of autoantibodies associated with the generation of pro-inflammatory cytokines as well as the generation of regulatory T cells through the mechanisms related to TGF- β inductors such as dendritic cells [50].

While SLE exemplifies a systemic autoimmune disorder, similar antigen-specific tolerance strategies have also been exploited in other, organ-targeted, autoimmune diseases. Rheumatoid arthritis (RA) is characterized by chronic inflammation of the synovial tissues and destruction of the joints because of immune interactions between autoreactive B and T cells and inflammatory factors. Citrullinated proteins are important autoantigens for many patients suffering from RA, especially in those with anti-citrullinated protein antibodies (ACPAs). Nevertheless, due to the variability of certain factors among the patients, any approach to vaccination in this regard should not be treated as universal. The second group of research is associated with the utilization of citrullinated antigens. For instance, the use of citrullinated multi-epitope antigens combined with rapamycin has proved beneficial in collagen-induced arthritis experiments leading to a decrease in pro-inflammatory cytokine secretion and to improved disease outcomes [51].

Other approaches can change immune responses to RA-related antigens. Antigenic liposomes utilizing Siglec receptor pathways and nanoparticles carrying rapamycin created antigen-specific B-cell tolerance and inhibited the development of arthritis in various models [52]. In the same way, the use of disease-related antigens together with calcitriol or some other metabolic modulator was shown to sustain regulatory immune responses and limit the Th1-associated or Th17-associated reactions [19,53,54].

The transition from successful preclinical studies to human ones has been difficult despite encouraging initial findings. Autoimmune diseases are highly heterogeneous, making it difficult to determine the proper autoantigens, immune pathways, and specific disease process. Difficult is also finding the right antigen and proper dose, selecting the appropriate adjuvants and delivery systems, and the heterogeneity of the diseases, makes it hard to predict treatment effectiveness. Moreover, the design of clinical studies is complicated because of the presence and frequent use of concurrent immunosuppressive treatments [55].

Overall, SLE and RA exemplify both the potential and the limitations of therapeutic vaccination in autoimmune diseases. In SLE, targeting disease-associated autoantigens, including nucleic acid-related and histone-derived antigens, may allow modulation of autoimmune responses without broad non-specific immunosuppression. In RA, the identification of disease-associated citrullinated proteins provides a rationale for antigen-specific approaches aimed at restoring immune tolerance. However, disease heterogeneity, the diversity of relevant autoantigens, and interindividual differences in immune responses remain major barriers to developing effective vaccines. Progress will therefore require appropriate patient stratification, precise identification of disease-related antigenic profiles, improved delivery systems, and carefully designed clinical trials [37,44,45].

## 4. Therapeutic Vaccines in Cancer

Therapeutic cancer vaccines are very exciting since their development enables the production of immune responses specific to tumor-associated antigens or tumor-specific antigens (the latter type of vaccines may have an even greater potential due to their ability to generate long-lasting immune memory). However, the effectiveness of these vaccines depends on many interrelated aspects, such as the choice of clinically significant and immunogenic antigens, the tumor mutation burden, the quality of antigen presentation, the specifics of the tumor immune microenvironment, and the timing of vaccination in relation to the surgery and other procedures, as well as the ability to overcome the effects of the tumor on the immune system.

Therapeutic vaccines enhancing the immune response against lung cancer cells have been investigated by Cortés-Jofré et al. [18]. The authors examined randomized controlled trials comparing therapeutic vaccines, either alone or in combination strategies, versus standard treatments or placebo in adults with advanced non-small cell lung cancer (NSCLC). They found no differences in overall survival, except for racotumomab, a murine monoclonal IgG1 antibody used as a vaccine targeting tumor gangliosides [56], which exhibited a small improvement in median survival time compared to placebo in a study with a small number of participants [57]. Moreover, randomized clinical studies evaluating TG4010, a viral vector vaccine expressing MUC1 (Mucin 1) and interleukin-2, have suggested a modest improvement in progression-free survival when combined with first-line chemotherapy in advanced NSCLC; however, the certainty of the evidence remains low, and no consistent overall survival benefit has been demonstrated [58]. Most of NSCLC overexpress epidermal growth factor receptor (EGFR) and its ligands. Phase II and III clinical studies evaluating CIMAvax-EGF as maintenance therapy after first-line platinum-based chemotherapy demonstrated an acceptable safety profile and suggested a modest survival benefit in selected patients; however, the magnitude of clinical benefit should be interpreted in light of study design and patient selection [59]. In the phase III ATALANTE-1 trial, these patients were randomly assigned to receive the multi-peptide vaccine, Tedopi (OSE-2101), or any other standard chemotherapy performed by investigators. Patients who received Tedopi experienced treatment-related adverse events; however, the trial did not prove the superiority of Tedopi [60]. The phase III randomized trial ARTEMIA, testing OSE-2101 against docetaxel chemotherapy in patients with advanced NSCLC who have become unresponsive to immunotherapy, is currently ongoing [61].

Immune-enhancing approaches have also been studied in patients with hepatocellular carcinoma (HCC), which is characterized by poor lymphocyte infiltration and, for this reason, is often unresponsive to immune checkpoint inhibitors. In HCC, available clinical evidence remains largely limited to early-phase studies. Phase I and II trials of peptide- and dendritic-cell-based vaccines have generally demonstrated acceptable tolerability and evidence of antigen-specific immune activation, but consistent improvements in objective tumor response, progression-free survival, or overall survival have not been established. Accordingly, the current evidence supports biological activity but remains insufficient to demonstrate a clinically meaningful benefit. Dendritic-cell-based vaccine can be either peptide-loaded DCs or tumor lysate-pulsed DCs. Peptide vaccines have mainly targeted tumor-associated antigens, such as alpha-fetoprotein (AFP), glypican-3 (GPC-3), and the human telomerase reverse transcriptase (hTERT)-derived peptide GV1001, whereas DCs have been loaded with AFP-derived peptides alone or in combination with other antigens (e.g., GPC-3), as well as tumor lysates [62,63]. Two peptide cocktails derived from GPC-3, WD-repeat-containing protein up-regulated in hepatocellular carcinoma (WDRPUH) and DNA glycosylase endonuclease VIII-like 3 (NEIL3) exhibited good tolerability and potential effectiveness against advanced HCC in two phase I studies [64].

Despite being the most clinically mature HCC vaccine approach, DC-based vaccines face significant challenges related to manufacturing complexity and high production costs. Currently, the most important progress has been reported in nanotechnology and exosome-based delivery platforms [65]. Wang et al. tested a nano-vaccine coated with exfoliated membrane from mature DCs after H22 (Hepatoma-22)-specific neoantigen stimulation, which remodeled tumor-associated neutrophils, induced strong T-cell responses, and suppressed both the primary tumor and distal tumor growth in mouse models [66]. Likewise, Zuo et al. designed a Dendritic-cell-derived exosome (DEX) vaccine, DEXP&A2&N, which enhanced the cross-presentation of tumor neoantigens and promoted tumor-specific T-cell responses, leading to tumor eradication in orthotopic HCC mice [67].

In recent years, growing attention has been directed to the development of HER-2 (Human epidermal growth factor receptor 2)-targeted vaccines in patients with breast cancer, who generally exhibit reduced spontaneous antibody production. Several immunogenic peptides deriving from different domains of HER-2 have been identified, including AE36, E75, and GP2. The E75 peptide, from the extracellular domain, elicits a robust cytotoxic T lymphocyte (CTL) response through its high affinity for the HLA-A2 and HLA-A3 molecules, while AE36, from the intracellular domain, can stimulate both CD4+ and CD8+ T cells. The four-amino-acid peptide LRMK may enhance vaccine immunogenicity by promoting the loading of MHC class II epitopes into the peptide-binding groove, thereby improving antigen presentation [68]. Moreover, CD27 agonism may improve both the effectiveness and the durability of vaccine-induced antitumor immune responses in HER2+ (Human epidermal growth factor receptor 2) breast cancer [69]. In human CD27 transgenic mice, the coadministration of a HER2 vaccine and an anti-CD27 agonist increased antigen-specific CD4+ T-cell responses, particularly long-lived memory cells, resulting in a higher rate of tumor regression compared with vaccination alone [70].

Early-phase clinical trials have consistently demonstrated acceptable safety and vaccine-induced immune responses, whereas evidence of durable clinical benefit remains limited. In addition, evidence regarding long-term safety and durability of vaccine-induced immune responses is still limited, and studies with larger populations and longer follow-up periods are necessary before a real-world implementation [71]. A multicenter, randomized, double-blind, placebo-controlled phase III trial of nelipepimut-S (NP-S) vaccine combined with granulocyte-macrophage colony-stimulating factor (GM-CSF) was conducted in 758 node-positive HER2-low-expressing breast cancer patients receiving adjuvant treatment. Injection site reactions were the most frequently reported AEs, and their rates were similar in the NP-S and placebo groups. After a median follow-up of 16.8 months, no statistically significant improvement in disease-free survival was observed in patients receiving NP-S vs placebo [72].

At present, research is being undertaken to develop therapeutic vaccines for colorectal cancer (CRC), particularly in recurrent and metastatic scenarios [73]. The existing clinical evidence is inconsistent. The Vigil vaccine has been evaluated only in a very limited number of patients, with preliminary evidence suggesting prolonged disease-free survival; [74]. OncoVAX has undergone phase III clinical testing, demonstrating positive impacts on 5-year overall survival among participants with stage II disease in comparison to those who underwent surgery alone. Previous phase II clinical trials have confirmed the tolerability and immunogenicity of OncoVAX in conjunction with chemotherapy. Other products, including VRP-CE A and the multi-peptide vaccine polyPEPI1018, have illustrated their immunological activity and sufficient safety; however, they should be tested in randomized trials in order to provide evidence of efficacy [75]. Among the virus-based platforms, the alphavirus vector VRP-CEA (virus-like replicon particle-carcinoembryonic antigen) has demonstrated a favorable immune modulation in stage III CRC, by expanding antigen-specific CD8+ effector memory T cells while simultaneously reducing regulatory T cells (Tregs) [76]. Moreover, the multi-peptide vaccine polyPEPI1018, targeting seven tumor-associated antigens, has been reported to be safe and to elicit specific immune responses when administered alongside maintenance chemotherapy for microsatellite stable (MSS) metastatic CRC. Nonetheless, further validation of these findings in randomized, controlled trials is necessary [77].

More recently, attention has been directed at induced pluripotent stem cells (iPSCs) as they share genetic profiles with malignancies and can train the immune system to recognize cancer cells. Interestingly, Jwo et al. found that an iPSCs vaccine, paired with CpG oligonucleotide as a vaccine adjuvant, had both prophylactic and therapeutic efficacy in CRC mouse models. Vaccination markedly enhanced CD8+ T-cell infiltration into the tumor microenvironment while T-cell depletion neutralized vaccine’s efficacy, confirming the role of these cells as drivers of antitumor responses. Through proteomic analysis two iPSCs-associated proteins—HNRNPU (heterogeneous nuclear ribonucleoprotein U) and NCL (nucleolin)—were identified as related to the antitumor immune response [78].

Wang et al. investigated the immunological impact, and the therapeutic potential of a DC vaccine pulsed with iPSC-derived exosomes (DC + EXO) in mouse melanoma models. Vaccination suppressed primary tumor progression and elicited a robust, durable T-cell response, preventing recurrence [79].

Yet, for melanoma there is a progressive shift from “passive killing” approaches to “active immunomodulation”. Among the platforms under investigation, mRNA vaccines are the most promising ones due to their rapid production cycles and their ability to be personalized, but peptide- and cell-based vaccines also continue to be explored. In melanoma, therapeutic vaccines are commonly investigated in combination therapies, often in association with immune checkpoint inhibitors (ICIs) such as PD-1 inhibitors. Through a synergistic approach, vaccines “prime” the immune system against tumor-specific antigens, whereas the ICIs “release the brakes” on T-cell activity to amplify the antitumor response [80].

The prototypical case is mRNA-4157. This is a patient-specific mRNA vaccine that contains up to 34 neoantigens. In resected, high-risk melanoma, clinical studies have indicated that using mRNA-4157 in addition to pembrolizumab improves disease-free survival when compared to those receiving only pembrolizumab, without significant safety concerns [81]. A recent study by Seclì et al. evaluated the efficacy of a viral vector multi-epitope neoantigen (nAg) vaccine in the B16F10 murine melanoma model, either before or after tumor resection, as monotherapy or in combination with a PD-1 inhibitor. They found that neoadjuvant nAg vaccination increases the protection against melanoma relapse, particularly when combined with checkpoint blockade, by enhancing the CD8+ T cell-dependent immunity [82].

Nano-enhanced vaccines might be helpful against metastatic melanoma. Melanoma-derived peptides capable of activating CD4^+^ T cells have been encapsulated in immunogenic nanoliposomes. For example, incorporation of the Toll-like receptors (TLR)4 agonist 3-deoxy-D-manno-octulosonic acid-lipid A (KDO2) has enabled the development of safe formulations that rapidly localize to secondary lymphoid organs to enhance peptide-specific immune responses [83]. Comparable progress has been achieved with nanoparticle-based mRNA vaccines, by co-delivering mRNA encoding immunostimulatory cytokines (e.g., IL-7, IL-12, and IFN-α) [84,85].

Despite these encouraging findings and a manageable safety profile, improvements in overall survival and progression-free survival are not yet consistent across cohorts [86].

Brain tumors remain among the most challenging malignancies to treat with immunotherapy, and several factors limit the potential efficacy of cancer vaccines. First, the blood–brain barrier (BBB) limits the penetration of many therapeutic agents into the brain. In addition, gliomas—the most common primary malignant brain tumors—typically shape an immunosuppressive tumor microenvironment, characterized by the recruitment and expansion of immunosuppressive cells (e.g., M2-polarized macrophages, Tregs, and myeloid-derived suppressor cells), which inhibit cytotoxic antitumor responses. Further challenges include tumor antigen heterogeneity and immune escape mechanisms [87]. To overcome these limitations, researchers are focusing on advanced delivery platforms to enhance antigen delivery across the BBB, combination approaches integrating vaccines with ICIs or cytokine modulators, and the development of multi-antigen vaccines to reduce the risk of immune evasion due to intratumoral heterogeneity [88]. In a multicenter phase III study, DCVax-L was evaluated in patients with glioblastoma in addition to standard-of-care treatment. The study reported longer survival compared with an external control population; however, the non-randomized design and use of an external comparator limit the strength of causal inference. [89]. Similar encouraging survival advantage has been documented in a 3-year follow-up phase II trial about DC vaccination in patients with newly diagnosed glioblastoma [90]. In a recent Italian study, an oncolytic Herpes Simplex Virus, R-115, engineered to target HER2 while expressing murine IL-12, has demonstrated robust cytotoxic and immunostimulatory activity in a glioblastoma mouse model. Notably, the approach induced direct oncolysis, eradicated secondary transplanted tumors, conferred long-term immunity, and exerted antitumor effects even against HER2 negative malignant cells, likely via antigen cross-presentation or epitope spreading [91].

Although prophylactic HPV (human papilloma virus) vaccination is expected to substantially reduce the incidence of HPV-related malignancies, these cancers will remain an important public health concern, particularly because vaccination does not eliminate established infections or pre-existing malignancies [92,93]. Consequently, there is a continued need for therapeutic HPV vaccines targeting high-risk HPV-associated lesions. These approaches primarily target the viral oncoproteins E6 and E7, which play central roles in HPV-mediated carcinogenesis. However, their relatively low intrinsic immunogenicity may limit effective immune recognition and cytotoxic T-cell responses, complicating the development of therapeutic vaccines [93,94,95]. Accordingly, strategies including codon optimization, electroporation, and nano plasmid delivery have been investigated to enhance DNA vaccine immunogenicity against HPV antigens [96,97].

Several DNA vaccines (GX-188E, VGX-3100, pNGVL4a-CRT/E7(detox), pNGVL4a-Sig/E7(detox)/HSP70, MEDI0457) have shown good safety and clinical activity [96]. [98]. There are multiple DNA vaccines being tested in early clinical studies that exhibit an acceptable level of safety and signs of clinical or immunological activity [99]. VGX-3100 is one of the most clinically progressed options, having successfully shown histological regression of cervical intraepithelial neoplasia (CIN) lesions associated with HPV16/18 in clinical patients. Here, the positive results show clinical effects, but it is not correct to conclude that the therapy will be equally efficient in the treatment of advanced HPV-positive malignancies [100].Considering that monotherapy may not provide long-term disease control, combinations of vaccination together with chemotherapy and/or immune checkpoint inhibitors are being tested. For instance, the usage of GX-188E along with pembrolizumab has been studied with the goal of improving the vaccine abilities to induce the activity of tumor-specific CD8^+^ T-cells [99,101]. Research on the use of replication-deficient viral vectors has also been undertaken for therapeutic vaccination against HPV-associated cancers. The strategy of using these types of viral vectors is based on the idea that they can boost the expression of pathogenic antigens and facilitate cellular anti-HPV responses. In preclinical studies, a synergetic approach combining therapeutic vaccination against HPV and PD-1/PD-L1 blockade has been proven to enhance antitumor immunity to a greater extent. In addition to viral vectors, development of protein vaccines, such as TVGV1 and peptide vaccines, has also been pursued. Researchers are also working on the next generation of vaccines that utilize mRNAs with HPV16 and HPV18-derived antigens. In the preclinical studies using the mHTV-03E2 vaccine, the combination of vaccine and immune checkpoint inhibitors proved to be effective by generating immunity and producing other beneficial effects [101,102]. HPV vaccination represents a cornerstone in primary cancer prevention, demonstrating high efficacy in preventing persistent oncogenic infections and subsequent dysplastic lesions. Beyond prophylactic applications, therapeutic vaccination strategies targeting HPV-driven antigens aim to stimulate cell-mediated immune responses against established malignancies. The vaccine mainly protects against two types of HPV, which are called HPV16 and HPV18. We need to make sure it works against other types of HPV as well. Importantly, we need to know if it really helps people who already have advanced cancer [96].

Notably, the development of therapeutic vaccines for chronic hepatitis B (CHB) has gained growing interest to minimize liver damage and prevent HCC development [103]. Nasvac (HeberNasvac) is a novel mucosal and parenteral therapeutic vaccine containing both hepatitis B surface antigen (HBsAg) and core antigen (HBcAg), designed to stimulate innate and adaptive immune responses. Nasvac has successfully completed preclinical development and multiple clinical trials. A randomized phase III clinical trial comparing Nasvac with pegylated interferon (Peg-IFN) in patients with CHB found greater viral suppression and liver-protective effects, while also demonstrating a more favorable safety profile than Peg-IFN [104,105]. Beyond this clinically evaluated approach, several next-generation vaccine platforms are being investigated preclinically. An LNP (lipid nanoparticles)-based mRNA vaccine encoding multiple HBV antigens (core, polymerase, and preS2-S) generated strong HBV-specific T-cell and antibody responses in mice and reduced circulating HBsAg, with a temporary decline in HBeAg in about half of treated animals. However, it did not significantly reduce intrahepatic viral replication or viremia, suggesting that further optimization or combination strategies are required [106]. DNA-based and vector vaccines are also being explored. The plasmid vaccine INO-1800 (± IL-12 adjuvant) generated HBV-specific T cells capable of targeting infected hepatocytes, whereas HB02 VAC-ADN induced CD4^+^ T-cell responses but was associated with viral rebound following antiviral discontinuation [107]. In another study, virus-like vesicle (VLV) hybrid viral vectors were engineered to enhance expression of the HBV middle surface antigen (MHBs) and stimulate strong antigen-specific CD8^+^ T-cell and antibody responses in mice [108].

Despite substantial progress in vaccine technology and antigen discovery, the clinical development of effective cancer vaccines remains challenging. Several interrelated factors may account for this discrepancy. Target antigens should be sufficiently tumor-enriched, appropriately expressed, processed, and presented by HLA molecules while minimizing the risk of immune-mediated damage to healthy tissues [109]. In addition, intratumoral antigenic heterogeneity and the presence of distinct tumor subclones may allow tumors to escape vaccine-induced immune responses. The tumor microenvironment can further restrict the activation and function of vaccine-induced T cells, while the timing of vaccination may substantially influence therapeutic efficacy. Finally, a robust vaccine-induced immune response does not necessarily translate into tumor regression or prolonged survival. Overall, effective cancer vaccination therefore depends on appropriate antigen selection and vaccine design, efficient T-cell trafficking, modulation of the tumor microenvironment, and rational combination therapies [110]. Clinical efficacy of therapeutic cancer vaccines has historically not been as high as predicted despite encouraging immunogenicity in various tumor types. The biological and clinical parameters that determine the efficacy of therapeutic vaccination do not merely reflect the platform used but rather a combination of interrelated factors. Most importantly, one of the key determinants is antigen selection. Traditional vaccine approaches are often intended to target TAAs with unsubstantiated tumor-specificity or immunogenicity, thus favoring immune escape and restricting long-lasting antitumor responses. Next-generation sequencing-based identification of patient-specific neoantigens has dramatically increased antigen specificity and is one of the major advances in personalized cancer vaccination [111].

The marked heterogeneity of malignant tumors represents another fundamental challenge. Interpatient and intratumoral heterogeneity creates unique antigenic landscapes, maintaining constant tumor evolution under immune-selective pressure. Consequently, immune responses induced by vaccines that are specific to a few epitopes may fail as the tumor expresses other mutations or loses expression of target antigens. Moreover, even if antigen presentation is successful, the vaccine-induced cytotoxic lymphocytes must also be able to infiltrate the tumor and remain functional within the tumor microenvironment [112].

The immunosuppressive tumor microenvironment is the other significant limiting factor for the efficacy of a vaccine. Despite producing measurable T-cell responses a vaccine may ultimately fail to block tumor growth because of active suppression by populations of Tregs, myeloid-derived suppressor cells, tumor-associated macrophages, and abundance of immunosuppressive cytokines or immune checkpoint pathways. These findings explain why many therapeutic vaccines elicit strong immunological responses but do not translate into improved progression-free or overall survival. Thus, immunogenicity cannot be considered as a surrogate marker of clinical efficacy [113].

Therapeutic cancer vaccines have yet to deliver meaningful clinical benefit when used alone, and it is becoming increasingly evident that they will be most effective when integrated as components of multimodal treatment strategies. Emerging strategies include combination with immune checkpoint inhibitors, optimizing the timing of vaccination to the minimal residual disease (MRD) state, and more specific neoantigen selection. Together, these advances support a shift from empirical vaccine development toward precision cancer vaccinology—wherein patient selection, antigen prioritization and tumor immune microenvironment modulation are viewed as equal contributors to therapeutic success [114].

## 5. Neurodegenerative Diseases

Vaccine-based approaches may contribute to the treatment of neurodegenerative disorders, such as Alzheimer’s disease (AD) and Parkinson’s disease (PD) by targeting pathological proteins, modulating chronic neuroinflammation, and inducing protective humoral responses. While vaccine development in oncology is much more developed, in neurodegeneration it remains in its early stages. The transition to clinical use is complicated by the blood–brain barrier, the need to ensure long-term safety, the uncertainty of the best pathology to target, and the difficulty of proving disease-modifying effects through clinically meaningful cognitive or motor endpoints [21].

Gene-based vaccines use DNA, RNA and/or viral vectors to supply genetic instructions directly to host cells, thus enabling the generation of target proteins and producing a long-lasting immune activation. Despite several challenges, earlier studies have shown promising results, including the ability to cross blood–brain barrier and ensure efficient delivery [115].

Virus-like particles (VLP)-based vaccines may help reduce protein accumulation in neurodegenerative disorders and slow disease progression, through their higher immunogenicity and safety, as compared to the traditional inactivated and live-attenuated vaccines [116]. For example, combining a non-toxic N-terminal fragment of amyloid-β (Aβ) with the hepatitis B core antigen led to consistent production of anti-Aβ antibodies in mice without triggering harmful T-cell inflammation. Additionally, Aβ fiber aggregation was reduced and nerve-like PC12 cells were protected from Aβ toxicity [117]. Immunization with the DNA vaccine pVAX1-IL-4/SYN-B in a mouse PD model resulted in robust anti- Alpha-synuclein (αS) antibody responses, increased IL-4 levels and tyrosine hydroxylase-positive neuronal counts, as well as motor improvements [118].

Other immunotherapeutic strategies may involve the development of protein-based vaccines. In AD, peptide vaccines (e.g., CAD106) have been shown to produce strong immune responses against amyloid-β (Aβ) plaques with minimal adverse effects [119]. On the other hand, in multiple sclerosis protein-based therapies targeting myelin-related antigens reduce autoimmune-mediated demyelination and promote immune tolerance. However, achieving a balance between immune suppression and disease control remains very challenging [21]. In its early-phase trials, the AADvac1 vaccine demonstrated an acceptable safety and immunogenicity profile, comprising a synthetic tau peptide linked to keyhole limpet hemocyanin (KLH) and produced with the adjuvant Alhydrogel [120,121]. In a randomized placebo-controlled phase II ADAMANT study, AADvac1 vaccination resulted in the production of antibodies against abnormal tau. Indeed, vaccine-induced antibodies could be detected in the cerebrospinal fluid with a concentration of approximately 0.3% of that which could be found in the serum, while other analyses revealed a decrease in phosphorylated tau and neurofibrillary lesions [111]. Although these outcomes confirm that the target was reached and the biological effect occurred, further investigation is needed to determine whether they were associated with cognitive improvements [122].

In synucleinopathies, the PD01A vaccine targeting α-synuclein provided long-lasting antibody responses and good tolerance in its Phase I study [123]. These results demonstrate that vaccination against α-synuclein is viable and effective, although it does not translate into clinical efficacy. Furthermore, research conducted in transgenic mouse models indicated that α-synuclein-targeting vaccines (PD01A and PD03A) could decrease pathological α-synuclein accumulation [121,124]. It must be pointed out that these preclinical results cannot be confused with early-phase clinical results that concentrated on safety and immunogenicity. 

Protein engineering has made the creation of modified Aβ oligomers possible, such as Amyloid-beta 42 conformationally constrained (Aβ42CC), which can initiate immune reactions against harmful oligomeric forms. ALZ-101 is a vaccine created by this method that is currently in phase I clinical trials. Preliminary results have shown that the vaccine is safe and effective. However, these early phase results mostly confirm only its viability, safety, and immune action, and it is unknown whether ALZ-101 will be able to slow down cognitive decline or halt the progression of the disease [125,126].

The UB-312 vaccine is a type of immunogenic peptide that generates antibodies against the harmful forms of oligomeric and fibrillar α-synuclein protein. The results of the preclinical studies indicate that the accumulation of α-synuclein protein is decreased and the motor and gastroenterological symptoms are improved in animal models [127]. In Phase I clinical trials of UB-312 application, the immune response against target substances was observed, as well as indications of reduced aggregated α-synuclein. In addition, the cerebrospinal fluid antibody response was correlated with motor function [128].

Alternatives to protein-based vaccines have been proposed, such as the Multi-Target Epitope Platform (Multi-TEP) combining the target antigen with multiple antigenic peptides, eliciting memory T helper cells and antibody production [129]. The DNA vaccine AV-1959D was designed to induce immunity against Aβ by combining Aβ1-11 with multiple T cell epitopes from tetanus, hepatitis B, and influenza antigens. In animal models, this vaccine triggered a robust cellular immune response against foreign epitopes, avoided autoreactivity, and induced strong anti-Aβ antibody production, paving the way for subsequent clinical evaluation [130]. In addition, RNA-based vaccines, such as the anti-Aβ AV-1959R and anti-tau AV-1980R, within immunogenic multi-TEP platforms elicited robust antibody responses against pathological forms of both Aβ and tau proteins in murine models [131,132].

Recent evidence suggests that the peripheral immune system plays a pivotal role in modulating inflammation within the central nervous system, particularly through dendritic cells, which are reduced and functionally impaired in certain neurodegenerative diseases [133,134]. Preclinical studies indicate that DCs can be used as natural adjuvants, promoting Th2-mediated immune responses and antibody production, as well as contributing to the clearance of intracellular protein aggregates and the enhancement of Treg activity. As a result, DC-based vaccines may promote glial cell polarization toward neuroprotective phenotypes but their safety and efficacy will have to be investigated in clinical trials [22,135].

## 6. Cardiovascular Diseases

Vaccine-based therapies are an increasingly attractive therapeutic strategy in the field of cardiovascular diseases.

Cardiovascular vaccines aim to target molecules produced by the body associated with disease, to regulate chronic vascular inflammation, or to provoke protective immune responses targeting pathways involved in atherosclerosis and cardiovascular remodeling. In pressure overload mouse models, a vaccine targeting Igfbp7 (insulin-like growth factor-binding protein 7) that is up-regulated in senescent murine hearts improved cardiac function. Researchers designed two peptides derived from the N-terminal region of murine Igfbp7, which were conjugated to KLH and administered with aluminum hydroxide as an adjuvant [23].

Koriyama et al. injected spontaneously hypertensive rats with a plasmid DNA vaccine encoding hepatitis B core antigen and angiotensin II (Ang II). The vaccine stimulated the production of anti-Ang II antibodies without triggering self-reactive T-cell activation and the immune response was sustained for over six months. Vaccinated rats experienced a reduction in systolic blood pressure and overall survival rates improved. Moreover, heart tissue analysis revealed a significant decrease in perivascular fibrosis. These findings suggest that Ang II DNA vaccines could provide a novel therapeutic option for long-term management of hypertension [136]. The combination of vaccines developed against the Ang II type 1 receptor (AT1R, using ATRQβ-001) and the α1D adrenergic receptor (α1D-AR, using ADRQβ-004) resulted in greater reductions in systolic blood pressure in mouse models compared to either of these vaccines alone. In addition, both vaccines combined had positive effects on vascular remodeling, cardiac hypertrophy, fibrosis and renal injury, as well as overall good tolerance [137,138]. However, ensuring the translation of these initially encouraging findings to humans requires long time and further research.

Beyond hypertension, dyslipidemia seems to be amenable to therapeutic vaccines. Preclinical studies have examined vaccines targeting apolipoprotein B100 (ApoB100), the main component of low-density lipoprotein (LDL), as well as cholesteryl ester transfer protein (CETP), which takes part in the exchange of cholesterol esters and triglycerides between high-density lipoprotein (HDL) and LDL [139]. However, in a phase I clinical trial evaluating a CETP-targeting vaccine [140] antibody production was insufficient [139]. Recently, Proprotein Convertase Subtilisin/Kexin Type 9 (PCSK9) has emerged as an important target of treating patients who suffer from severe dyslipidemia. A PCSK9-targeted vaccine has been developed and was shown to induce robust and sustained antibody responses in animal models. Kawakami et al. created a peptide-based formulation of small mouse PCSK9 peptides conjugated with KLH. The combination of these peptides with an adjuvant, when administered to male apolipoprotein E-deficient mice, resulted in sustained anti-PCSK9 antibody generation in the absence of T-cell activation, improved lipoprotein profiles, a reduction in total plasma cholesterol and Very-low-density lipoprotein (VLDL) levels, and increased expression of the LDL receptor on the surface of cells [141]. Fukami et al. focused on a novel therapeutic target for dyslipidemia, angiopoietin-like protein 3 (ANGPTL3), involved in the metabolism of LDL and triglycerides. In experimental mouse models, a peptide vaccine against ANGPTL3 improved lipoprotein profile and dyslipidemia-associated conditions, such as atherosclerosis and fatty liver disease [142].

Researchers have recently developed an antiplatelet vaccine targeting S100A9/CD36 (S100 calcium-binding protein A9/cluster of differentiation 36) signaling in platelets, involved in arterial thrombosis, to prevent recurrent stroke and related complications. Immunization with this vaccine resulted in long-lasting anti-S100A9 antibodies and significantly reduced thrombus formation in rats, with effects comparable to clopidogrel, and without increasing bleeding risk [143].

In recent years, RNA technologies explored during the COVID-19 pandemic have been considered in the cardiovascular field. RNA-based therapies such as small interfering RNAs (siRNAs) and microRNA (miRNA), together with gene-editing approaches, are currently being evaluated to control cardiovascular risk factors and eventually support the repair of damaged heart tissue [139,144]. For instance, researchers developed an mRNA vaccine against circulating heat shock protein 60 (HSP60) abnormally expressed in heart failure, that is being associated with inflammatory signaling and apoptosis [24]. Despite high expectations, RNA-based therapies have yet to fulfill the promise and further preclinical and clinical research is necessary.

**Figure 1:**
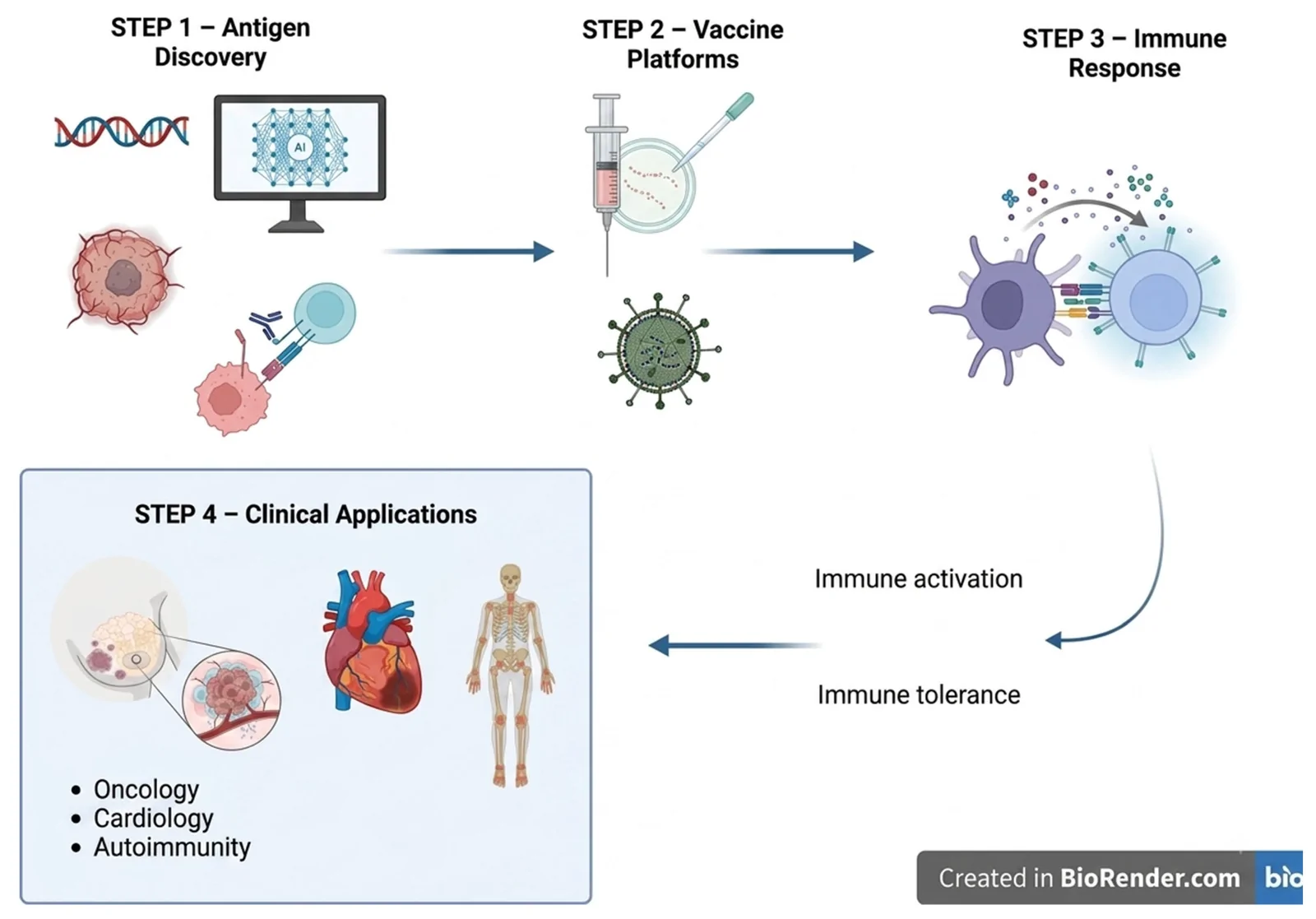
Conceptual framework of personalized vaccine development for non-communicable diseases. Step 1 (Antigen Discovery): Identification and selection of disease-specific neoantigens/targets via genomic sequencing and AI/bioinformatic pipelines. Step 2 (Vaccine Platforms): Incorporation of identified candidates into delivery platforms (e.g., nucleic acids, viral vectors, or cell-based formulations). Step 3 (Immune Response): Antigen presentation by antigen-presenting cells (APCs, purple) to T cells (blue), accompanied by cytokine signaling, driving targeted immune modulation. Step 4 (Clinical Applications): Downstream therapeutic outcomes directed toward immune activation or immune tolerance across major non-communicable disease areas, including oncology, cardiology, and autoimmune disorders. Blue solid arrows indicate the sequential progression of the workflow.

Figure 1 provides an overview of the conceptual workflow underlying personalized vaccine development, from antigen discovery to clinical application, highlighting the dual potential for immune activation and immune tolerance.

## 7. Allergic Diseases

Allergic diseases are an important category of non-infectious diseases in which antigen-specific immunotherapeutic procedures have the potential to develop towards precision vaccinology. Allergen immunotherapy (AIT) is currently the only method of treatment that can cause long-term immunity against the allergens causing the disease. However, conventional AIT requires the use of natural allergen extracts, which may differ in composition from batch to batch, take a long time to process, and may be accompanied by adverse systemic reactions. Thus, in recent years, the emphasis in research has been shifted to the use of the so-called vaccine approaches based on molecularly defined allergens and rationally designed formulations of the allergens aimed at improved safety and treatment standardization [145,146].

Currently, there are many new types of methods being studied, such as recombinant allergens, allergen derivatives that can reduce allergic reactions, VLP, and vaccines based on nucleic acids. The goal of all these methods is to maintain the immune epitope necessary for tolerance induction and influence IgE-mediated allergy reactions. For example, methods based on peptides can specifically target T-cell epitopes of allergens without triggering IgE-mediated cross-linking and activation of mast cells or basophils. However, recombinant allergens and hypoallergenic allergens could be developed to keep the necessary T-cell epitope and to decrease their affinity to allergen-specific IgE. These changes in the immune response could lead to the transformation of immunopathogenic type 2 immune response into regulatory responses, increased production of anti-inflammatory cytokines (such as interleukin-10 and Transforming growth factor-beta), and the development of specific blocking antibodies against allergens (mostly different IgG4) [145,146].

The recent development of VLP allergen immunotherapy offers an innovative option for creating vaccine-like drugs. VLPs may present allergenic epitopes in an immunogenic and well-defined form to trigger an allergen-specific IgG response, which could minimize the activation of IgE-related effector cells. Therefore, this technique could achieve a great deal of immunogenic effectiveness as with current vaccine technology, combined with the characteristics typical for AIT [147]. At the same time, the use of mRNA technologies, which help achieve a temporary use of allergen-derived antigen, becomes particularly interesting. It was demonstrated in a series of preclinical experiments using lipid nanoparticles with mRNA coding important dust mite allergens that it is possible to reduce allergic airway inflammation in animal models, [148].

The rapidly developing science of molecular allergology can lead to personalized treatment and diagnosis of allergic disorders. Component-resolved diagnostics (CRD) can detect molecular allergen components that cause allergies and separating true sensitization from cross-reactivity. This will allow for the selection of specific allergens that should be tested on the patient instead of utilizing standard allergen extracts. In this situation, personalized allergy vaccines can be developed based on the allergens determined by the examination, major allergens, type IgE pattern of reactivity, and other essential immunological features. The method can help researchers select recombinant allergens, peptide epitopes, VLP-based products, or nucleic acid formulations [149].

However, despite obvious progress in this area, transferring personalized vaccines into practice is notoriously complicated. The main difficulties originate from the great diversity in allergic sensibility, frequent overlapping of many allergens, as well other unknow factors.

## 8. Conclusions

Vaccines are rapidly evolving from providing protection against infectious diseases to being employed in the treatment and prevention of many non-infectious (or non-communicable) diseases. The rapid advancements in the fields of immunology, genomics, bioinformatics, and biomaterials science are allowing the development of new innovative vaccine platforms that have specific immune responses or restore immune tolerance, depending on the disease mechanisms.

The most advanced clinical development is in the field of oncology with the emergence of personalized neoantigen-based vaccines and the integration of these vaccines with immune checkpoint inhibitors. In addition, there is an increasing amount of preclinical and early clinical data demonstrating the potential benefits of tolerogenic vaccines for individuals suffering from autoimmune diseases, as well as immunotherapeutic strategies targeting pathological proteins associated with neurodegenerative and cardiovascular diseases. Continued advancement of mRNA technologies, nanoparticle delivery systems, dendritic cell vaccines, and next-generation adjuvants, are accelerating the translation of vaccine-based approaches to precision medicine.

However, there are still significant barriers to widespread clinical application, including disease heterogenicity, poor identification of target antigens, complex fabrication, stringent regulations, lengthy duration of safety studies, and variability in immune response between individuals. In addition, current evidence indicates that vaccines are unlikely to be successful if used alone and will be of greater clinical benefit as part of multi-modality treatment strategies that incorporate immunotherapy, targeted therapies, and advanced genomic profiling.

Future innovations predict a convergence of systems biology with AI-assisted antigen discovery, high-throughput sequencing technology, and custom manufacturing platforms to allow for the creation of truly personalized immunotherapies based on the molecular and immunological profile of each individual patient.

Personalization in this regard must be viewed in a more complex way, as a process with multiple aspects rather than just as choosing an antigen for a patient. Future vaccines will increasingly involve individual molecular profiling, immune phenotype determination, computational antigen engineering, individualized vaccine manufacturing, and long-term immune monitoring. This means that in the future it will be possible to adjust vaccine composition and treatment assemblages on the go according to the changes in the disease and based on patient-specific immune reactions.

While there are still many scientific and translational hurdles to overcome, personalized vaccines are positioned to play a central role in the advancement of disease prevention, immune enhancement, and long-term clinical management for a variety of non-communicable diseases.

## Figures and Tables

**Table 1:** The main vaccine platforms and their underlying mechanisms, advantages, and current limitations.

Platform	NCDs Application	Core Mechanism	Key Advantages	Main Limitations	References
Protein/Peptide vaccines	Cancer, autoimmune diseases, neurodegenerative diseases, allergic diseases	Direct antigen presentation via APCs → mainly humoral ± CD4^+^ responses	Well-characterized, safe, scalable	Low immunogenicity, requires adjuvants	[1,10]
DNA vaccines	Cancer, autoimmune diseases, neurodegenerative diseases, cardiovascular diseases	In situ antigen expression following nuclear delivery	High stability, ease of production	Low transfection efficiency, limited immunogenicity	[6]
mRNA vaccines	Personalized cancer vaccines; emerging applications in autoimmune, neurodegenerative, cardiovascular, and allergic diseases	Cytoplasmic translation → strong humoral and cellular responses	High efficacy, rapid and flexible design	Stability issues, reactogenicity	[2,3]
saRNA/circRNA	Primarily cancer and emerging applications in chronic inflammatory and other NCDs	Prolonged intracellular antigen expression	Dose sparing, sustained immune stimulation	Limited clinical validation, complex manufacturing	[11]
Viral-vector vaccines	Cancer, chronic viral infections with oncogenic potential, and selected immune-modulatory applications	Efficient gene delivery → robust T-cell Priming	Strong CD8^+^ responses,	Pre-existing immunity, safety concerns	[7]
Dendritic-cell vaccines	Personalized cancer immunotherapy and selected tolerogenic approaches in autoimmune diseases	Repetitive antigen display → B-cell activation	Highly immunogenic, no genetic material	Limited cellular immunity	[5]
Nanoparticle-based vaccines	Cancer, autoimmune diseases, neurodegenerative diseases, cardiovascular diseases, and allergic diseases	Ex vivo antigen-loaded APCs activate T cells	Personalized, potent cellular responses	Costly, labor-intensive	[12]
VLP-based vaccines	Cancer, neurodegenerative diseases, allergic diseases, and selected autoimmune applications	Enhanced antigen delivery and targeting	Improved stability and biodistribution	Formulation complexity, potential toxicity	[13,14]
Exosome-based vaccines	Cancer, particularly personalized neoantigen delivery and tumor microenvironment modulation	Innate immune activation (e.g., Toll-like receptor (TLR), Stimulator of interferon genes (STING)	Tailored immune responses, increased efficacy	Reactogenicity, safety balance	[15]

**Table 2:** Overview of personalized vaccination strategies across major non-communicable diseases. The table summarizes the principal therapeutic targets, preferred vaccine platforms, current stage of clinical development, and major challenges associated with each disease category.

Disease Category	Main Targets	Vaccine Platforms	Development Stage	Key Challenges	Key References
Cancer	Neoantigens, TAAs, viral oncoproteins	mRNA, peptide vaccines, dendritic cell vaccines, viral vectors, nanoparticles	Preclinical to phase III clinical trials	Tumor heterogeneity, Immune escape, manufacturing complexity	[4,18]
Autoimmune diseases	Autoantigens (DNA, histones, citrullinated proteins, myelin antigens)	Tolerogenic peptides, nanoparticles, liver-targeted delivery, dendritic cell-based approaches	Preclinical to early clinical development	Maintaining antigen-specific tolerance while preserving immune competence	[19,20]
Neurodegenerative diseases	Amyloid-β, tau, α-synuclein	Peptide vaccines, DNA/RNA vaccines, VLPs, dendritic cell vaccines	Preclinical to phase II clinical trials	Blood–brain barrier penetration, long-term safety, efficacy validation	[21,22]
Cardiovascular diseases	PCSK9, ApoB100, ANGPTL3, Ang II, Igfbp7	Peptide vaccines, DNA vaccines, mRNA-based approaches	Mainly preclinical, limited clinical data	Long-term efficacy, target validation, safety assessment	[23,24]

## Data Availability

No new data were created or analyzed in this study. Data sharing is not applicable to this article.
